# Evaluation of China’s medical and older adult care integration policy based on the PMC model

**DOI:** 10.3389/fpubh.2025.1660238

**Published:** 2025-08-29

**Authors:** Yahui Ba, Zhonghua Luo

**Affiliations:** ^1^School of Health Management, Gansu University of Chinese Medicine, Lanzhou, Gansu, China; ^2^School of Marxism, Gansu University of Traditional Chinese Medicine, Lanzhou, Gansu, China

**Keywords:** medical and older adult care integration policy, PMC index model, aging, policy evaluation, text mining

## Abstract

**Objective:**

Many countries are experiencing above-average levels of aging, and older adults have higher demands on healthcare and older adult care systems. It is important for governments to improve policies to enhance the quality of healthcare and older adult care systems. However, research on policies combining healthcare and older adult care remains limited. This study evaluates healthcare and older adult care policies, particularly the medical and older adult care integration policy, to provide a reference for policy interventions that can achieve sustainable development.

**Methods:**

This study introduced a policy maturity and consistency (PMC) index model based on text mining technology to analyze 28 representative medical and older adult care integration policy documents. The analysis model identifies policy deficiencies and provides actionable insights for improving future policy frameworks.

**Results:**

The PMC indices of the 28 policies ranged from 7.67 to 4.40, with 22 policies rated as ‘good’ and 6 as ‘acceptable.’ The policy texts demonstrated strong standardization and scientific rigor in terms of content expression and technical structure. However, regarding specific performance, 16 policies scored above the average, while 12 scored below the average. Analysis of the PMC surface plots for the 28 policies reveals that the overall three-dimensional surface exhibits a noticeable concave feature, indicating that the implementation potential and effectiveness of nearly half of the policies still have significant room for improvement.

**Conclusion:**

Through the analysis of China’s medical and older adult care integration policy texts using the PMC model, it was concluded that China’s medical and older adult care integration policies have a high degree of systematicity and consistency. The following recommendations are proposed: improve the policy system to enhance policy coverage and inclusiveness; optimize the combination of policy tools to enhance policy implementation flexibility; and improve the diversified security mechanism to enhance the systematic support and precise supply capabilities of medical and older adult care integration services.

## 1 Introduction

Global aging is accelerating at an unprecedented rate, and it is now an indisputable fact that the international community is rapidly entering an aging era (1, 2). Significant differences exist in the degree of aging among different countries and regions (3), with East Asia and Europe being particularly prominent (4). By the end of the 1970s, the global population aged 65 and above was predicted to exceed the population under 18 for the first time. By 2,100, the older adult population may reach nearly 11 billion, a historical peak. This demographic shift, characterized by an aging population, profoundly affects social mechanisms (5), economic systems, and sustainable development paths, and places higher demands on governments to improve their medical and older adult care service systems (6). Therefore, evaluating medical and older adult care integration policies not only helps clarify effective policy intervention pathways and better leverage the role of policies in integrating medical resources and older adult care services to improve service efficiency, but also provides essential support for achieving healthy aging and sustainable social development.

Currently, scholars have conducted in-depth research on medical and older adult care integration policies from multiple perspectives. In terms of research methods, the vast majority of studies are based on the perspective of policy tools, analyzing the problems and shortcomings of medical and older adult care integration policies from the viewpoint of supply, demand, and environment, and proposing optimization paths based on this analysis (7–10). Regarding research content, Min Zhang and other scholars have studied the issue of insufficient funding for developing medical and nursing integration policies (11). In contrast, Chao Rong and other scholars have conducted in-depth discussions on improving the capabilities of nursing staff in medical and nursing integration institutions (12). Regarding research scope, scholars such as Xiaoming Su focused on a specific region (13), with the research scope being a small urban community. In contrast, scholars such as Mengya JIANG conducted comparative analyses between areas (14), comparing the medical and older adult care integration policies of Xinjiang and Sichuan provinces in China.

A multidimensional analysis of medical and nursing care integration policies enables us to gain a more comprehensive understanding of the strengths and weaknesses of these policies during their implementation. In particular, applying qualitative research methods allows us to interpret policy texts at a deeper level, helping to reveal the underlying logic and motivations behind policies. However, existing research has mostly remained at the macro level, neglecting the comparability of policy texts and between policies. At the same time, there are still certain limitations in terms of methodology. Overall, there is a lack of systematic, long-term, and comprehensive quantitative analysis of medical and nursing care integration policies, with issues such as a narrow scope of research and a short period. The PMC Index Model is a policy analysis method focusing on in-depth quantitative analysis of policy texts, effectively reducing subjective bias. It provides a comprehensive review of policies through multi-angle evaluation and clearly illustrates policies’ advantages and potential shortcomings using intuitive PMC surface diagrams.

As one of the most populous countries in the world, China is facing profound challenges posed by rapid population aging. The fast pace and broad scope of its aging process are typical globally. It has become a prominent representative of aging issues in East Asia and one of the most representative countries in terms of the global aging trend. Against this backdrop, how to effectively respond to the dual pressures of medical care and older adult care brought about by aging has become an essential issue of common concern to the government and society. To this end, this study takes the integrated medical and older adult care policies issued by China between 2015 and 2025 as its research object, uses the ROSTCM6.0 text mining software to construct a PMC index model, and establishes a scientific and systematic quantitative evaluation system for integrated medical and older adult care policies. Through in-depth analysis and comprehensive evaluation of policy content, this study aims to reveal the characteristics and shortcomings of current policies, thereby providing theoretical support and practical reference for China and other countries in formulating and improving policies related to the integration of medical care and older adult care.

## 2 Materials and methods

### 2.1 Data sources

In 2013, the State Council of China first proposed ‘actively promoting the integration of medical care and older adult care services.’ Still, it did not explicitly use the term ‘medical and older adult care integration.’ In 2015, China issued its first national-level policy specifically targeting medical and older adult care integration, formally introducing the concept of ‘medical and older adult care integration’ in an official document for the first time. Therefore, as of the start of this study, we collected policy documents related to medical and older adult care integration in China from January 2015 to April 2025, using the keywords ‘medical and older adult care integration,’ ‘older adult care,’ and ‘medical and older adult care integration’ for the search. Policy texts were primarily sourced from the following three channels: first, the Chinese Government Website^1^; second, ‘Peking University Law Database’^2^; and third, literature searches, from which relevant policies were extracted.

Inclusion Criteria: ① The policy documents must be closely related to integrated healthcare and older adult care, directly stipulating or reflecting content related to integrated healthcare and older adult care. ② Policy types include laws and regulations, plans, opinions, and other government policy documents. ③ The policies included must be at the national level, i.e., policies on integrated healthcare and older adult care issued by the State Council, the General Office of the State Council, departments under the State Council, and agencies directly under the State Council. This is because policies issued at the national level serve as the starting point and basis for local policy formulation, possessing guidance, authority, and foresight. ④ Exclusion criteria: First, policies that have been abolished; second, policy interpretations, news media reports, and other such documents; third, documents that duplicate those already included in the policy text repository. After careful reading and screening, 28 policy texts were ultimately included in the policy text repository, as detailed in Table 1.

**Table 1:** Twenty eight policies on integrating medical care and older adult care.

Code	Policy name	Publication Date
P1	Notice of the General Office of the State Council on Issuing the Outline of the National Health Care Service System Plan (2015–2020)	2015.03.06
P2	Notice of the General Office of the State Council Forwarding the Guidance Opinions of the National Health and Family Planning Commission and Other Departments on Promoting the Integration of Medical and Health Care Services with Older Adult Care Services	2015.11.18
P3	Notice of the State Council on Issuing the Outline of the Strategic Plan for the Development of Traditional Chinese Medicine (2016–2030)	2016.02.22
P4	Notice of the General Office of the National Health and Family Planning Commission on Issuing the Division of Responsibilities for Key Tasks in Integrating Medical Care and Older Adult Care	2016.04.01
P5	Notice of the General Office of the National Health and Family Planning Commission and the General Office of the Ministry of Civil Affairs on Issuing the Division of Responsibilities for Key Tasks in Integrating Medical Care and Older Adult Care	2016.04.07
P6	Notice of the Ministry of Civil Affairs and the National Development and Reform Commission on Issuing the 13th Five-Year Plan for the Development of Civil Affairs	2016.06.24
P7	The Central Committee of the Communist Party of China and the State Council issued the ‘Healthy China 2030’ Planning Outline.	2016.10.25
P8	Opinions of the General Office of the State Council on Comprehensively Opening Up the Older Adult Care Service Market and Improving the Quality of Older Adult Care Services	2016.12.07
P9	Notice of the State Council on Issuing the 13th Five-Year Plan for the Development of the Older Adult Care Service and the Construction of the Older Adult Care System	2017.02.28
P10	Opinions of the General Office of the State Council on Promoting the Development of Older Adult Care Services	2019.03.29
P11	Notice on Doing a Good Job in the Approval and Registration of Medical and Nursing Care Institutions	2019.05.27
P12	Several Opinions on Deepening the Development of Medical Care and Older Adult Care Integration	2019.10.23
P13	Notice of the General Office of the National Health Commission, the General Office of the Ministry of Civil Affairs, and the Office of the National Administration of Traditional Chinese Medicine on Issuing the Service Guidelines for Medical and Older Adult Care Integration Institutions (Trial)	2019.12.23
P14	Notice from the Office of the National Health Commission, the Office of the Ministry of Civil Affairs, and the Office of the National Administration of Traditional Chinese Medicine on the Issuance of the Management Guidelines for Medical and Older Adult Care Integration Institutions (Trial Version)	2020.02.17
P15	Notice from the Office of the National Health Commission on the Selection of the First Batch of Pilot Institutions for Remote Collaborative Services Combining Medical Care and Older Adult Health Care	2020.08.28
P16	Notice from the Office of the National Health Commission, the Office of the Ministry of Civil Affairs, and the Office of the National Administration of Traditional Chinese Medicine on the Issuance of the Management Guidelines for Medical and Older Adult Care Integration Institutions (Trial Version)	2020.09.27
P17	Opinion of the Ministry of Housing and Urban–Rural Development, the National Development and Reform Commission, the Ministry of Civil Affairs, the National Health Commission, the Medical Insurance Bureau, and the National Committee on Aging on Promoting the Development of Home-Based and Community-Based Older Adult Care Services by Property Service Enterprises	2020.11.24
P18	Notice on Launching a Quality Improvement Campaign for Medical and Nursing Care Institutions	2020.12.03
P19	Opinions of the General Office of the State Council on Promoting the Healthy Development of Older Adult Care and Childcare Services	2020.12.14
P20	Notice from the Ministry of Industry and Information Technology, the Ministry of Civil Affairs, and the National Health Commission on the Issuance of the ‘Action Plan for the Development of the Smart Health and Aging Industry (2021–2025)’	2021.10.20
P21	Opinions of the Central Committee of the Communist Party of China and the State Council on Strengthening Aging Work in the New Era	2021.11.18
P22	Notice of the State Council on Issuing the 14th Five-Year Plan for the Development of the National Aging Cause and the Older Adult Care Service System	2021.12.30
P23	Notice on Launching an Initiative to Enhance Community Medical and Older Adult Care Integration Capabilities	2022.03.23
P24	Guiding Opinions on Further Promoting the Development of Medical Care and Older Adult Care Integration Issued by the National Health Commission, National Development and Reform Commission, Ministry of Education, Ministry of Civil Affairs, Ministry of Finance, Ministry of Human Resources and Social Security, Ministry of Natural Resources, Ministry of Housing and Urban–Rural Development, Ministry of Emergency Management, State Administration for Market Regulation, and National Medical Insurance Bureau	2022.07.18
P25	Notice on Promoting Typical Experiences from Pilot Programs Combining Medical Care and Older Adult Care	2023.03.14
P26	Notice on the Issuance of the Guidelines for Home-based and Community-based Medical and Nursing Care Services (Trial Version)	2023.11.01
P27	Guiding Opinions on Promoting High-Quality Development of Medical and Older Adult Care Integration Services Issued by the National Health Commission, the Ministry of Civil Affairs, the National Medical Insurance Bureau, the National Administration of Traditional Chinese Medicine, and the National Center for Disease Control and Prevention	2024.12.12
P28	Opinions of the Central Committee of the Communist Party of China and the State Council on Deepening the Reform and Development of Older Adult Care Services	2024.12.30

### 2.2 Research methods

**Figure 1:**
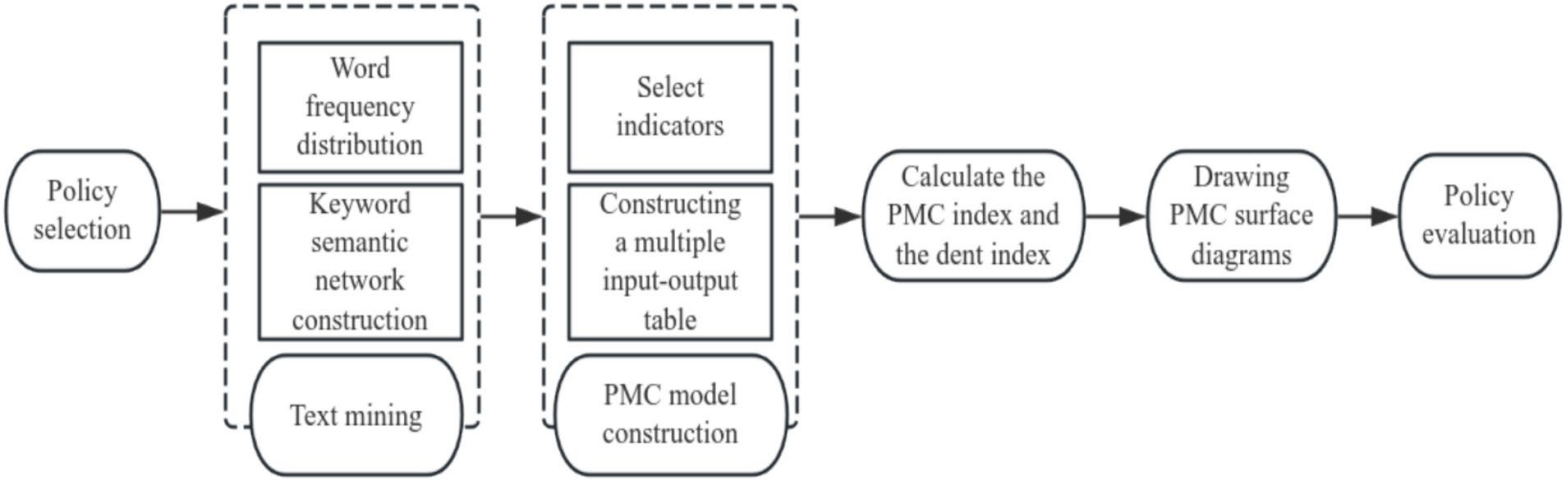
Policy research process.

The PMC index model is a quantitative policy analysis and evaluation method based on the ‘Omnia Mobilis’ hypothesis proposed by Estrada in 2011 (15). This assumption holds that all factors are interrelated and dynamically changing, and that the importance of any variable cannot be ignored. The PMC model typically includes 9 to 10 primary variables and several secondary variables, with consistent weights for each secondary variable under each primary variable. The PMC index model focuses on the quantitative analysis of policy texts. It determines variable parameters through policy text mining and assigns strict values based on policy content, thereby reducing subjective bias in variable extraction. The results are presented intuitively through PMC surface diagrams, which are suitable for evaluating and comparing single or multiple policies, as shown in Figure 1.

## 3 Research process

### 3.1 Text mining

This paper utilizes the ROSTCM6 text mining software to compile the policy texts of 28 selected policies into TXT files. Then it performs in-depth analysis and word frequency statistics on these 28 policy texts. The preprocessing steps for text mining include: (1) text cleaning, i.e., deleting irrelevant metadata (such as ‘document number,’ ‘issuing department,’ and ‘issuing time’), followed by word segmentation; (2) Stopword removal, i.e., filtering out common particles, pronouns, and prepositions that have no semantic weight in policy analysis. Based on the standard stopword list built into ROSTCM 6.0, a custom list of domain-independent terms was added, such as ‘should,’ ‘of,’ and ‘on.’ Next, stem extraction was performed, and the top 100 high-frequency words were retained; (3) Part-of-speech (POS) tagging, which means using the software’s built-in POS parser to identify nouns, verbs, and adjectives related to policy components (e.g., ‘objectives ‘and’ measures’), while deduplicating standardized terms (e.g., unifying ‘system’ and “mechanism” into a single term).

After preprocessing, the software generates a list of the top 30 high-frequency keywords, as shown in Table 2. These are then mapped to the initial evaluation indicators. For example, high-frequency terms such as ‘Service’ (2,945 occurrences) and ‘Pension’ (1,740 occurrences) directly inform the inclusion of variables such as X8 (policy objectives) and X6 (policy target).

**Table 2:** High-frequency keyword list.

Code	Vocabulary	Frequency	Code	Vocabulary	Frequency	Code	Vocabulary	Frequency
1	Service	2,945	11	Integrated Medical and Older Adult Care	512	21	Traditional Chinese Medicine	330
2	Pension	1740	12	Community	503	22	Planning	307
3	Institution	1,690	13	Country	392	23	Organization	306
4	Medical	1,151	14	System	376	24	Resources	299
5	Health	1,076	15	Personnel	375	25	Nursing	295
6	Older Adult	1,034	16	Protection	371	26	Rehabilitation	295
7	Hygiene	1,031	17	Regime	371	27	Hospital	294
8	Society	656	18	Age	364	28	Safety	270
9	Construction	628	19	Facilities	351	29	Technology	240
10	Management	608	20	Policy	330	30	Education	230

**Figure 2:**
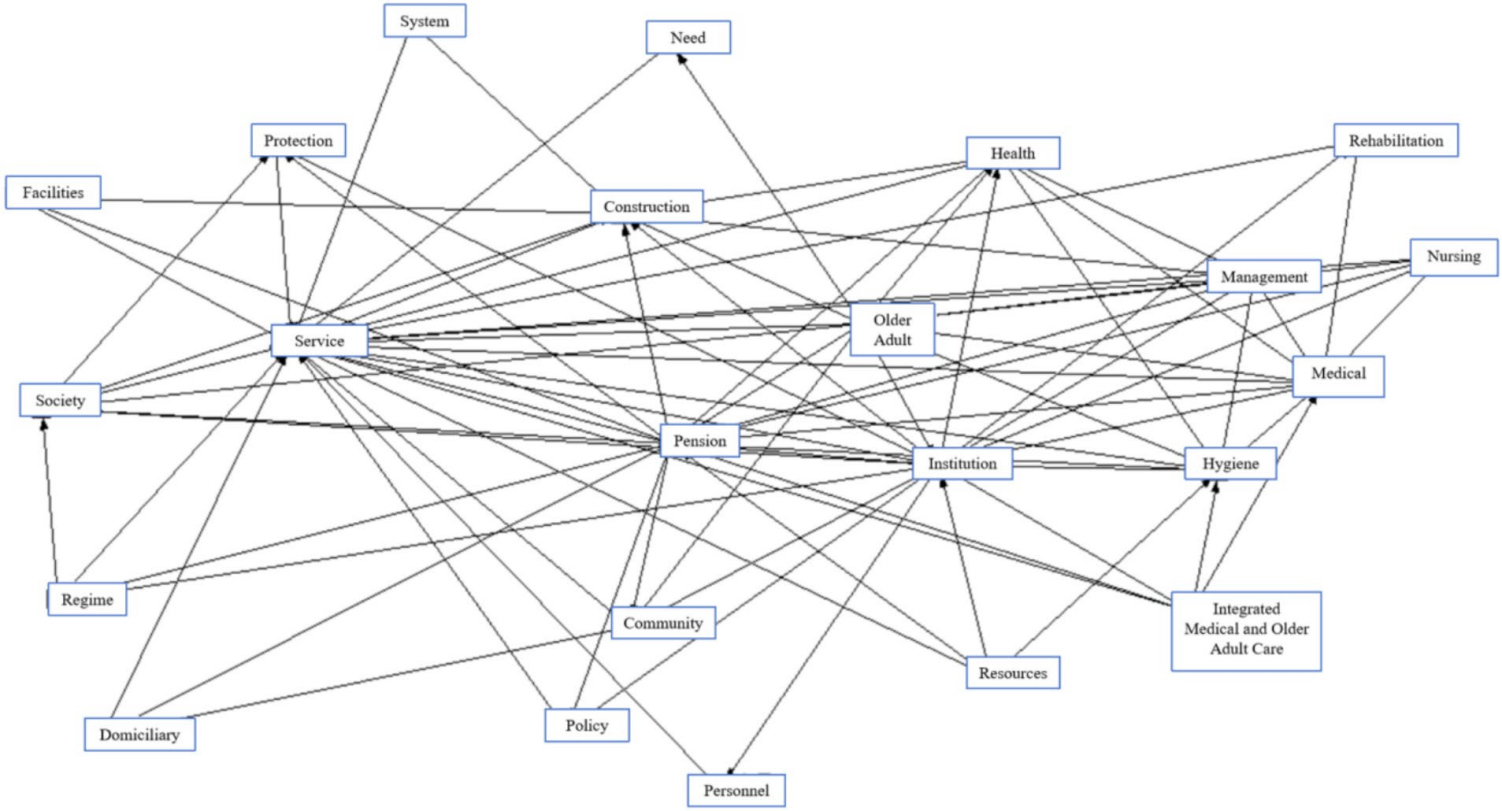
Semantic network diagram of high-frequency words.

Subsequently, based on the high-frequency keywords extracted, we used the ROSTCM6 software to draw a semantic network diagram, as shown in Figure 2. In this semantic network, the core nodes in the chart’s middle include ‘Pension,’ ‘Service,’ ‘Medical,’ and ‘Institution,’ with the remaining keywords distributed around these core concepts. The number of connections between keywords reflects the closeness of their relationships and the strength of their associations. Notably, keywords such as ‘Service,’ ‘Institution,’ ‘Medical,’ and ‘Hygiene’ highlight key areas where further strengthening of connections is needed within the policy framework. Therefore, when constructing a multi-input scale, priority should be given to these aspects’ in-depth integration and reinforcement.

### 3.2 Construction of the PMC index model

Based on the results of high-frequency keyword analysis and semantic network diagram analysis obtained using ROSTCM6 software, and taking into account the specific characteristics of the medical and older adult care integration policy, this paper makes preliminary settings for policy evaluation variable indicators by comprehensively referring to the settings proposed by Mario Arturo Ruiz Estrada and other scholars in existing literature (16–23). After clarifying the connotation of the first-level variables, the selection of second-level variables considered various influencing factors. The importance and weights of the second-level variables are the same. The values of the second-level variables follow a distribution of [0,1] and take the values 0 or 1. When the policy content includes or involves the relevant variable, the value is 1; if it is unrelated to the variable, the value is 0. Variable X10 is merely an indicator of whether the policy is open; therefore, no second-level variables were set. After expert discussion and revision, the PMC Integrated Medical and Older Adult Care Policy Variable Evaluation Index Model was established, which includes 9 first-level variables and 39 s-level variables, as detailed in Table 3.

**Table 3:** Policy evaluation variable indicators.

First-level variable	Second-level variable	Evaluation scale	Source or basis
X_1_ Policy nature	X_1:1_Forecast	Whether the policy involves forecast content: 1 if yes, 0 if no	Sun YC et al. (17)
	X_1:2_Supervision	Whether the policy involves supervisory content: 1 if yes, 0 if no	
	X_1:3_Recommendation	Whether the policy involves the recommended content: 1 if yes, 0 if no	
	X_1:4_Support	Whether the policy involves support measures: 1 if yes, 0 if no	
	X_1:5_Guide	Whether the policy involves guiding content: 1 if yes, 0 if no.	
	X_1:6_Description	Whether the policy involves the described content: 1 if yes, 0 if no.	
X_2_ Policy effectiveness	X_2:1_Long-term	1 if in force>5 years,0 otherwise	
	X_2:2_Mid-term	1 if in force for 3 to 5 years, 0 otherwise	
	X_2:3_Short-term	1 if in force<3 year, 0 otherwise	
X_3_ Issuing agency	X_3:1_State Council and its constituent departments	1 if the policy is issued by the State Council and its constituent departments, 0 otherwise	Wang Y et al. (20)
	X_3:2_National Health Commission	1 if the policy is issued by the National Health Commission, 0 otherwise	
	X_3:3_Other agencies or departments	1 if the policy is issued by other agencies or departments, 0 otherwise	
X_4_ Policy instruments	X_4:1_Supply-driven	1 if the policy involves service-related supply-side policy tools, 0 otherwise	
	X_4:2_Environmental type	1 if the policy involves planning and other environmental policy tools, 0 otherwise	
	X_4:3_Demand-driven	1 if the policy involves demand-driven policy tools such as guidance, 0 otherwise	
X_5_ Policy content	X_5:1_Platform development	1 if the policy involves platform development, otherwise 0	Based on Zhong H et al. (23).
	X_5:2_Talent development	1 if the policy involves talent development, otherwise 0	
	X_5:3_Improved systems	1 if the policy involves improved systems, otherwise 0	
	X_5:4_Service supervision	1 if the policy involves service supervision, otherwise 0	
	X_5:5_Safeguards	1 if the policy involves safeguards, otherwise 0	
X_6_ Policy target	X_6:1_Administrative department industry	1 if the policy involves administrative departments industry, otherwise 0	
	X_6:2_Medical institution	1 if the policy involves medical institutions, otherwise 0	
	X_6:3_Older adult care facility	1 if the policy involves older adult care facility, otherwise 0	
	X_6:4_Social organizations	1 if the policy involves social organizations, otherwise 0	
	X_6:5_Health industry	1 if the policy involves the health industry, otherwise 0	
X_7_ Policy evaluation	X_7:1_Well-founded	1 if the policy is well-founded, otherwise 0	Cao N et al. (18)
	X_7:2_Clear objective	1 if the policy is clear objective, otherwise 0	
	X_7:3_Detailed planning	1 if the policy is detailed planning, otherwise 0	
	X_7:4_Scientific approach	1 if the policy is scientific approach, otherwise 0	
X_8_ Policy objectives	X_8:1_Promoting industrial development	1 if the policy promotes industrial development, otherwise 0	Based on Li W et al. (22)
	X_8:2_Improving the quality of older adult care medical and nursing care needs	1 if the policy is to improve the quality of older adult care medical and nursing care needs, otherwise 0	
	X_8:3_Improvements in medical care	1 if the policy is to improve medical standards, otherwise 0	
	X_8:4_Promoting policy coordination	1 if policys promote policy coordination, otherwise 0	
	X_8:5_Meet medical and nursing care needs	1 if the policy meets medical and nursing care needs, otherwise 0	
X_9_ Policy guarantees	X_9:1_Policy guarantees	1 if the policy involves policy guarantees, otherwise 0	Based on Li Y et al. (21)
	X_9:2_Talent cultivation	1 if the policy involves talent cultivation, otherwise 0	
	X_9:3_Financial support	1 if the policy involves financial support, otherwise 0	
	X_9:4_Departmental collaboration	1 if the policy involves departmental collaboration, otherwise 0	
	X_9:5_Supervision and evaluation	1 if the policy involves supervision and evaluation, otherwise 0	
X_10_ Policy disclosure	No secondary variables		

A multi-input–output table is constructed based on policy evaluation variable indicators, with variable scores assigned in binary [0,1] form. This enables a multidimensional measurement and quantitative policy content analysis as shown in Table 4.

**Table 4:** Input–output table.

First-level variable	X_1_	X_2_	X_3_	X_4_	X_5_	X_6_	X_7_	X_8_	X_9_	X_10_
Second-level variable	X_1:1_	X_2:1_	X_3:1_	X_4:1_	X_5:1_	X_6:1_	X_7:1_	X_8:1_	X_9:1_	
	X_1:2_	X_2:2_	X_:32_	X_4:2_	X_5:2_	X_6:2_	X_7:2_	X_8:2_	X_9:2_	
	X_1:3_	X_2:3_	X_3:3_	X_4:3_	X_5:3_	X_6:3_	X_7:3_	X_8:3_	X_9:3_	
	X_1:4_				X_5:4_	X_6:4_	X_7:4_	X_8:4_	X_9:4_	
	X_1:5_				X_5:5_	X_6:5_		X_8:5_	X_9:5_	
	X_1:6_									

### 3.3 Measurement of PMC index and indentation index

According to Estrada’s PMC index model, the calculation method is as follows: First, assign equal weights to all secondary variables in the input–output table. According to Equation 1, if a specific integrated medical and older adult care policy aligns with a given secondary variable, its parameter is set to 1; otherwise, it is set to 0. Then, calculate the value of a specific primary variable according to Equation 2. Finally, the PMC index is obtained by summing all primary variables according to Equation 3. The policy indentation index is calculated by subtracting the PMC index from the sum of nine primary variables and secondary variables according to Equation 4.


(1) X={XR:[0,1]}

(2) $$X_{T}=\sum_{j=1}^{N}\frac{X_{t_{j}}}{T(X_{t})},t=1,2,3$$

(3) $$\mathrm{PMC}=[\begin{array}{c} X_{1}(\sum_{j=1}^{6}\frac{X_{1j}}{6})+X_{2}(\sum_{j=1}^{3}\frac{X_{2j}}{3})\\ +X_{3}(\sum_{j=1}^{3}\frac{X_{3j}}{3})+X_{4}(\sum_{j=1}^{3}\frac{X_{4j}}{3})\\ +X_{5}(\sum_{j=1}^{5}\frac{X_{5j}}{5})+X_{6}(\sum_{j=1}^{5}\frac{X_{6j}}{5})\\ +X_{7}(\sum_{j=1}^{4}\frac{X_{7j}}{4})+X_{8}(\sum_{j=1}^{5}\frac{X_{8j}}{5})\\ +X_{9}(\sum_{j=1}^{5}\frac{X_{9j}}{5}) \end{array}]$$

(4) Indentation index=9−PMC

According to the PMC rating standards indicated by Estrada (15) and the relevant adjustments made by scholars such as Peixin Duan (24), these policies are classified as excellent, good, acceptable, and poor, as shown in Table 5.

**Table 5:** Policy evaluation criteria for the PMC index.

PMC Index	Evaluation	Policy indentation index	Evaluation
8.00–9.00	Excellent	0.00–1.00	Low depression
6.00–7.99	Good	1.01–3.00	Central depression
4.00–5.99	Acceptable	3.01–5.00	Acceptable level of indentation
0–3.99	Poor	5.01–9.00	Unacceptable level of indentation

### 3.4 Construction of PMC surface diagrams

To present the strengths and weaknesses of integrated medical and older adult care policies across various dimensions more clearly and intuitively, it is necessary to create a three-dimensional surface diagram based on the results of the PMC index calculation. In the PMC surface diagram, convex surfaces represent variables with higher scores, indicating a higher policy level; concave surfaces represent variables with lower scores, indicating a lower policy level and considering the symmetry and balance of the surface when plotting, since all policy documents are public policies, X10, which has no secondary variables, was excluded. This paper plots the PMC surface diagram based on the following 3 × 3 matrix.


$$\mathrm{PMC}=[\begin{array}{ccc} X_{1} & X_{2} & X_{3}\\ X_{4} & X_{5} & X_{6}\\ X_{7} & X_{8} & X_{9} \end{array}]$$

## 4 Research results and discussion

### 4.1 Calculation of the PMC index

Based on the aforementioned indicator system, the primary variables were calculated to determine the PMC index for each policy. The results are presented in Table 6. The average PMC index for the 28 integrated medical and older adult care policies was 6.47, indicating that the policy indicators were generally good overall. Specifically, 22 policies are rated as ‘good’ and six as ‘acceptable.’ Among these, Policy P22 has a PMC index score of 7.67, ranking first, with an evaluation of ‘good,’ and a concavity index of 1.33, indicating a moderate concavity level. This suggests that the policy has a high overall quality and was formulated scientifically and reasonably; Policy P26 has a PMC index score of 4.4, rated as ‘acceptable,’ with a concavity index of 4.4, indicating an acceptable concavity level. This suggests that the policy is generally sufficient for integrated healthcare and older adult care content, but has some shortcomings and requires further improvement.

**Table 6:** 28 medical and nursing care integration policy PMC index scores.

Ranking	Code	X_1_	X_2_	X_3_	X_4_	X_5_	X_6_	X_7_	X_8_	X_9_	PMC Index	Evaluation	Policy indentation index	Evaluation
1	P22	1.00	0.33	0.33	1.00	1.00	1.00	1.00	1.00	1.00	7.67	Good	1.33	Central depression
2	P9	1.00	0.33	0.33	1.00	1.00	1.00	1.00	1.00	0.80	7.47	Good	1.53	Central depression
3	P25	1.00	0.33	0.33	1.00	1.00	1.00	1.00	1.00	0.80	7.47	Good	1.53	Central depression
4	P27	0.83	0.33	0.67	1.00	1.00	0.80	1.00	1.00	0.80	7.43	Good	1.57	Central depression
5	P20	0.83	0.33	0.67	1.00	0.80	1.00	1.00	1.00	0.80	7.43	Good	1.57	Central depression
6	P2	1.00	0.33	0.33	0.67	1.00	1.00	1.00	1.00	1.00	7.33	Good	1.67	Central depression
7	P4	0.67	0.33	0.33	1.00	1.00	1.00	1.00	1.00	1.00	7.33	Good	1.67	Central depression
8	P7	0.67	0.33	0.33	1.00	1.00	1.00	1.00	1.00	1.00	7.33	Good	1.67	Central depression
9	P28	0.83	0.33	0.33	1.00	1.00	1.00	1.00	0.80	1.00	7.30	Good	1.70	Central depression
10	P24	0.50	0.33	1.00	0.67	1.00	1.00	1.00	0.80	0.80	7.10	Good	1.90	Central depression
11	P10	0.67	0.33	0.33	1.00	0.80	1.00	1.00	0.80	1.00	6.93	Good	2.07	Central depression
12	P21	0.67	0.33	0.33	1.00	1.00	1.00	1.00	0.80	0.80	6.93	Good	2.07	Central depression
13	P17	0.50	0.33	1.00	1.00	0.80	1.00	1.00	0.80	0.40	6.83	Good	2.17	Central depression
14	P19	0.67	0.33	0.33	0.67	1.00	1.00	1.00	1.00	0.80	6.80	Good	2.20	Central depression
15	P1	0.67	0.33	0.33	0.67	1.00	0.60	1.00	1.00	1.00	6.60	Good	2.40	Central depression
16	P3	0.83	0.33	0.33	0.67	1.00	1.00	1.00	0.80	0.60	6.57	Good	2.43	Central depression
17	P5	0.83	0.33	0.33	0.67	0.80	0.80	1.00	1.00	0.60	6.37	Good	2.63	Central depression
18	P6	0.67	0.33	0.33	1.00	0.80	0.80	0.75	1.00	0.60	6.28	Good	2.72	Central depression
19	P16	0.50	0.33	0.33	0.67	1.00	1.00	1.00	0.80	0.60	6.23	Good	2.77	Central depression
20	P8	0.50	0.33	0.33	1.00	1.00	0.60	0.75	0.60	1.00	6.12	Good	2.88	Central depression
21	P23	0.67	0.33	0.33	0.67	0.60	0.80	1.00	0.80	0.80	6.00	Good	3.00	Central depression
22	P18	0.67	0.33	0.33	0.67	1.00	0.60	1.00	0.80	0.60	6.00	Good	3.00	Central depression
23	P12	0.50	0.33	0.33	0.67	0.80	1.00	0.75	0.60	0.80	5.78	Acceptable	3.22	Acceptable
24	P11	0.50	0.33	0.33	0.33	0.60	0.80	0.75	0.80	0.60	5.05	Acceptable	3.95	Acceptable
25	P13	0.33	0.33	0.33	0.67	0.40	0.60	1.00	0.80	0.40	4.87	Acceptable	4.13	Acceptable
26	P14	0.50	0.33	0.33	0.33	0.60	0.60	1.00	0.60	0.40	4.70	Acceptable	4.30	Acceptable
27	P15	0.50	0.33	0.33	0.33	0.40	0.60	1.00	0.80	0.40	4.70	Acceptable	4.30	Acceptable
28	P26	0.33	0.33	0.33	0.33	0.60	0.67	1.00	0.40	0.40	4.40	Acceptable	4.60	Acceptable
	Average	0.67	0.33	0.40	0.77	0.86	0.87	0.96	0.85	0.74	6.47	-	2.53	Central depression

**Figure 3:**
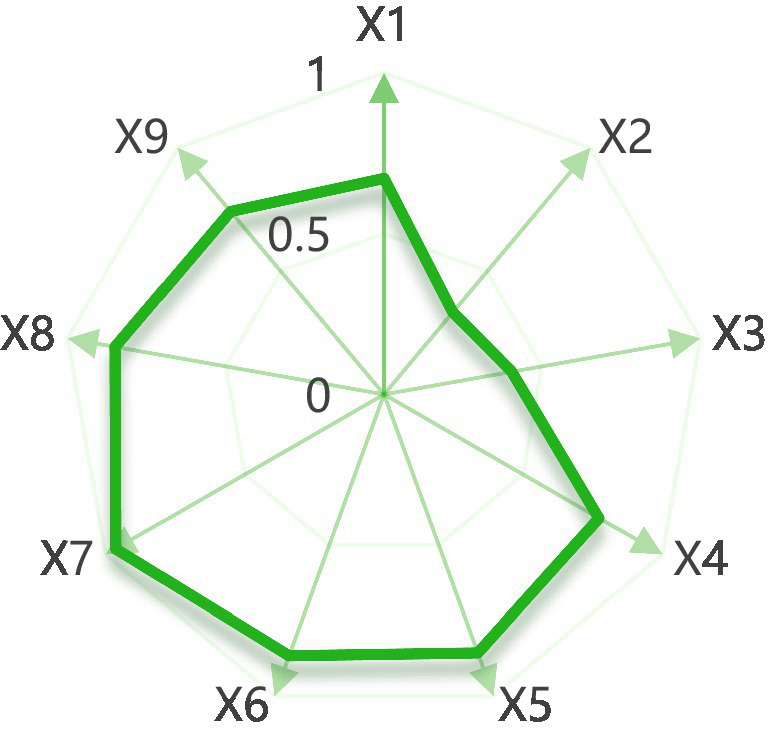
PMC mean radar chart.

The radar chart of the average PMC index values under the integrated medical and older adult care policy (see Figure 3) provides a more intuitive reflection of the overall completeness of the current policy. Except for indicators X_2_ (policy effectiveness) and X_3_ (issuing authority), the average values of all other indicators exceed 0.6, meeting the ‘good’ grade criteria. It should be noted that X_2_ and X_3_, due to their single-value characteristics (i.e., non-continuous variables), cannot directly reflect the overall quality of the policy based on their numerical values alone. Therefore, based on the performance of various indicators in the comprehensive radar chart, the overall level of China’s medical and older adult care integration policies is relatively high, and the content of the policies is becoming more extensive. It is worth noting that among the 28 policies, 16 policies have a PMC index greater than the average. In comparison, 12 policies have a PMC index less than the average, with nearly half of the policies falling below the average. Possible reasons include that some policies were enacted too early or are no longer fully applicable due to exceptional circumstances, such as policies issued during the COVID-19 pandemic. Additionally, policies enacted in the past 5 years are more comprehensive in all aspects compared to earlier policies, resulting in a higher average value. In contrast, policies issued earlier tend to score slightly below the average.

To further explore the impact of the COVID-19 pandemic on policies, this study grouped policies into pre-pandemic and post-pandemic categories for comparison. Policies enacted before December 2019 were assigned to the ‘pre-pandemic’ group, while those enacted on or after December 2019 were assigned to the ‘post-pandemic’ group. The results are presented in Table 7. The results show that the mean score for pre-pandemic policies was 6.55, while the mean score for post-pandemic policies was 6.40. However, the median score was higher for post-pandemic policies (6.80 vs. 6.60), with a larger standard deviation (1.03 vs. 0.77), a higher maximum value (7.67 vs. 7.47), and a lower minimum value (4.40 vs. 5.05). This indicates that post-pandemic policy quality exhibits a polarized trend: on one hand, higher-quality policies have emerged, while on the other hand, policies with poorer design quality have also appeared, resulting in overall volatility that increases, reflecting the complexity and challenges of policy formulation under emergency response conditions.

**Table 7:** Comparison between before and after the COVID-19 pandemic.

Statistics	Pre-pandemic (*n* = 12)	Post-pandemic (*n* = 16)
Mean	6.55	6.4
Median	6.6	6.8
Standard deviation (SD)	0.77	1.03
Minimum value	5.05	4.4
Maximum value	7.47	7.67

### 4.2 PMC surface diagram

**Figure 4:**
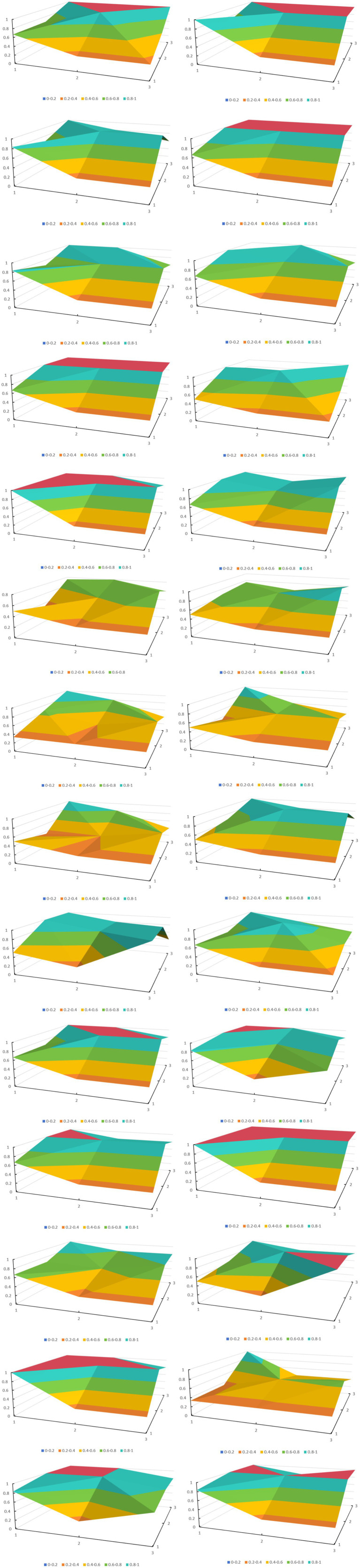
PMC surface diagram of 28 medical and nursing care integration policies.

This study constructed a three-dimensional matrix to plot the PMC surface diagram based on the first-level variable values of policy texts X_1_ to X_9_. The policy tools were spatially deconstructed using a graphical display format based on a three-dimensional coordinate system to provide a visual display method for policy optimization. The PMC surface diagrams of the 28 policies were laid out in order from left to right and from top to bottom, as shown in Figure 4.

The PMC surface plot of the 28 policies reveals their weaknesses and strengths. Overall, these policies perform poorly in the X_2_ and X_3_ indicators, while the average scores for the remaining indicators all exceed 0.6, meeting the criteria for a ‘good’ rating. The overall level of China’s integrated healthcare and older adult care policies is relatively high. Therefore, the X2 policy effectiveness and X3 issuing authority indicators, which have single-value characteristics, are excluded from discussion. Although the policies achieve a ‘good’ rating in the X_1_ indicator, they are relatively lacking compared to other indicators. The X_1_ (policy nature) aspect indicates that medical and older adult care integration policy documents could be further strengthened.

Due to space limitations, this study selected three representative PMC surface diagrams for analysis. The highest-scoring policy P22, the lowest-scoring policy P26, and the middle-scoring policy P19 were selected. Policy P22, ‘Notice of the State Council on Issuing the 14th Five-Year Plan for the Development of the National Aging Cause and the Older Adult Care Service System,’ (25) received the highest score, with a PMC value of 7.67 and a rating of ‘good.’ All indicators of Policy P22 are well-developed, indicating that this policy has high feasibility and applicability in the context of integrated medical and older adult care policies. This is because the policy was recently enacted and is part of a medium- to long-term plan, making it comprehensive in terms of policy nature, content, and target audience. It also employs many policy tools, has an extensive and reasonable policy evaluation, sets specific policy objectives, and provides adequate policy safeguards. The policy with the lowest score, P26 ‘Notice on Issuing the Guidelines for Home-based and Community-based Medical and Nursing Care Services (Trial Version) (26), had a PMC value of only 4.40, which is considered acceptable. All other indicators were below average except for the PMC value of policy X7, which was higher than the average. This indicates that the policy for integrating medical care and older adult care is well-designed in terms of policy evaluation, with detailed planning and clear objectives. However, there are some shortcomings. First, the policy is only a trial policy, and it was promulgated relatively early, resulting in a relatively limited range of policy tools. Second, the policy lacks detailed explanations of its content, objectives, and safeguards. There is no detailed information on platform construction, talent cultivation, or other related matters, and there are no detailed policy objectives. Furthermore, there is a lack of description of the supporting safeguards. Policy P19, ‘Opinions of the General Office of the State Council on Promoting the Healthy Development of Older Adult Care and Childcare Services,’ (27) scored in the middle range, with a PMC index of 6.80. Except for the indicators of X_3_ issuing agency and X_4_ policy tools, which were below average, all other indicators were above or equal to the average. This policy emphasizes achieving the goals of promoting industrial development and improving the quality of older adult care by providing more policy guarantees and continuously improving platform construction, talent cultivation, and service supervision. The shortcoming is that the policy tools are too limited.

## 5 Conclusion

### 5.1 Summary

This study surveyed 28 Chinese government policy documents on integrated medical and older adult care services and conducted policy evaluations based on a quantitative integrated medical and older adult care policy evaluation model and text mining. The following conclusions were drawn:

1. From the perspective of overall policy design, China’s medical and older adult care integration policy documents are highly systematic and consistent. According to the calculation results, the PMC index of the 28 policies ranges from 7.67 to 4.40, with 22 policies rated as ‘good’ and six as ‘acceptable,’ indicating that the policy documents are highly standardized and scientific in content expression and technical structure. However, in terms of specific performance, only 16 policies scored above the average. In contrast, 12 scored below the average, indicating that the implementation potential and effectiveness of nearly half of the policies still have significant room for improvement. Further analysis revealed that the scores for indicators such as the effectiveness of Policy X_2_, the level of the issuing authority for Policy X_3_, and the nature of Policy X_1_ were relatively low, reflecting shortcomings in the scope of application, enforcement strength, and institutional design of some policies. In response to this issue, the state should remain the core entity responsible for policy formulation, strengthening coordination mechanisms between the central and local governments, and improving the execution and response efficiency of relevant provincial departments in the policy implementation. In addition, policy formulation should focus on expanding the scope of policy coverage in terms of descriptive content and functions such as forecasting, regulation, guidance, and support. At the same time, the national government needs to formulate differentiated incentive measures that align with national conditions and maintain a degree of dynamic adjustment capability to enhance the adaptability and flexibility of policies. Through the design of incremental incentive mechanisms, it will be possible to stimulate the enthusiasm of local governments, market entities, and social forces, thereby comprehensively improving the implementation efficiency of medical and nursing care integration policies and better meeting the needs of an aging society.
2. There is still significant room for improvement in the current policy framework for integrating medical and older adult care. The PMC surface diagram analysis of 28 policies shows that the three-dimensional surface has a relatively noticeable concave feature. Except for the indicators X_2_ policy effectiveness and X_3_ issuing agency, the surface diagram shows a prominent concave feature in the three key dimensions of policy nature (X_1_), policy tools (X_5_), and policy guarantees (X_7_). Specifically, the scope of policy application is relatively limited, and there is a lack of systematic regulation of diversified older adult care models. Policy tools are limited in variety, and there is insufficient coordination between demand-driven and environment-driven tools. The policy support mechanism has not yet formed a multi-dimensional support system, and there is still room for improvement in areas such as financial investment, talent development, and quality supervision. To this end, it is recommended that policy optimization be promoted from three aspects: first, expand the scope of policy application to include non-institutionalized service models such as home-based older adult care and community-embedded older adult care into the policy framework; second, enrich the policy toolkit to emphasize the comprehensive use of diverse means such as economic incentives, information services, and technology promotion; third, improve the policy support system to establish a comprehensive support mechanism covering funding, talent cultivation, standardization, and quality supervision, thereby achieving a systematic upgrade of the policy system.
3. The degree of policy refinement still needs further improvement. Currently, most policy documents focus on direct factors such as the construction of medical and nursing care facilities, establishment of service systems, and training of professional personnel. However, there are still obvious shortcomings in responding to the diverse needs of older people and improving the accuracy of services. The essence of promoting the integration of medical care and older adult care lies in the deep integration of healthy aging and high-quality older adult care services. Therefore, in the policy-making process, greater emphasis should be placed on responding to the actual needs of the older adult population, taking into full consideration indirect influencing factors such as ‘older adult people’s preferences for older adult care,’ ‘health status,’ ‘accessibility and efficiency of services,’ and ‘family care capacity.’ For example, establishing a service matching mechanism based on the health grading of older people can enhance resource allocation’s precision and service provision’s effectiveness. Alternatively, guiding the development of community-embedded medical and older adult care services can strengthen the medical support capabilities for home-based older adult care, driving a policy shift from an ‘institution-centered’ approach to a ‘people-centered’ one. Only by comprehensively considering both direct service provision and indirect influencing factors can policies’ scientific rigor, targeting, and practicality be truly enhanced, thereby driving the development of integrated medical and older adult care services toward greater precision, personalization, and sustainability.

### 5.2 Recommendations

In response to the current issues of insufficient coverage in specific areas and regional imbalances in China’s medical and older adult care integration policies, the following countermeasures and recommendations are proposed to meet better the growing demand for medical and older adult care integration among the older adult in the context of accelerating population aging:

1. Improve the policy framework and enhance policy coverage and inclusiveness.

Based on the Chinese government’s current policy documents, we have found that the government is still planning from a macro perspective. Still, it is difficult to comprehensively cover micro-level situations such as implementing medical and older adult care integration policies in various localities from a macro perspective alone. The scope of policy application can be expanded to include non-institutionalized service forms such as home-based older adult care and community-based older adult care in the policy support system, promote the decentralization of medical and older adult care resources, and enhance the service capabilities of grassroots organizations. Additionally, when issuing policies, they could be tailored by region or province, with policy frameworks developed based on the specific circumstances of each area, to provide more precise and more explicit guidance for the formulation of local policies.

2. Optimize the policy toolkit and enhance policy implementation flexibility.

Based on an assessment of China’s current medical and older adult care integration policy documents, it was found that the policy documents are weak in terms of policy tools. Supply-side, demand-side, and environment-side policy tools should be combined to enhance the policy’s flexibility and adaptability, encourage local areas to explore innovative policy measures, and form a virtuous cycle of ‘central guidance and local pilot programs.’

3. Improve diversified security mechanisms and enhance medical and nursing care integration services’ systemic support and precision supply capabilities.

To lay a solid foundation for implementing medical and older adult care integration policies, a multi-dimensional support system covering financial support, talent cultivation, and quality supervision should be established. At the same time, demand-oriented approaches and digital technology empowerment should be strengthened to promote the development of a refined and intelligent service system. On the one hand, the government needs to increase fiscal investment, establish a special funding support mechanism, strengthen the coordinated training and career development path design of older adult care and medical nursing personnel, and establish a service quality supervision system covering the entire chain to improve industry standardization and normalization. On the other hand, in the process of policy formulation and implementation, greater emphasis should be placed on the individual differences and actual needs of the older adult, exploring a tiered and categorized service system based on health status, self-care ability, and service preferences, and achieving a transition from ‘uniform provision’ to ‘tailored approaches.’ In addition, digital technologies such as artificial intelligence, big data, and telemedicine should be actively introduced to improve resource allocation efficiency and service response capabilities, promote the in-depth development of smart health and medical care integration, and thereby build a solid, responsive, efficient, and people-oriented medical and nursing care integration support system.

### 5.3 Shortcomings and further work

This study systematically evaluated integrated medical and older adult care policies based on the PMC index model, providing new theoretical perspectives and methodological approaches for policy analysis in related fields. However, there are still several limitations: (1) The study assumes equal weights for all PMC dimensions, which may overlook their differential impacts on policy effectiveness. Future work should test the robustness of results through sensitivity analyses and alternative weighting methods. (2) This study focuses on policy texts at the national level and has not yet explored policy implementation at the local level or regional differences in depth. The next step could be to refine the research focus to individual provinces and municipalities, comparing the similarities and differences between different regions in terms of policy design, implementation methods, and implementation effects, thereby providing a basis for constructing a medical and older adult care integration policy system that is more adaptable to local conditions. (3) The PMC index has some limitations that are worth considering. Due to the inherent limitations of the policy evaluation model, current research struggles to fully reflect the complexity and dynamic changes of policies in real-world implementation. Its assumption of equal weights may overlook the differing levels of importance across various dimensions. At the same time, the binary scoring system (0/1) simplifies the complexity of policy content and fails to capture partial implementation scenarios. Future research could enhance robustness by integrating complementary methods (such as multi-criteria decision analysis (MCDA) for weighted assessment or the Delphi method for expert validation), thereby achieving methodological triangulation and gaining deeper insights.

## Data Availability

Publicly available datasets were analyzed in this study. This data can be found at: https://www.gov.cn/, https://www.pkulaw.com/law. Further inquiries are available from the corresponding author on reasonable request.

## Footnotes

^1^ https://www.gov.cn/

^2^ https://www.pkulaw.com/law
